# Knowledge, Attitude, and Practice Among Caregivers About Lumbar Puncture in the Pediatric Age Group in the Western Region of Saudi Arabia

**DOI:** 10.7759/cureus.87925

**Published:** 2025-07-14

**Authors:** Amira A Nemri, Salha S Asiri, Fahad F Mansouri

**Affiliations:** 1 Department of Pediatrics, King Abdulaziz University, Jeddah, SAU

**Keywords:** caregivers, knowledge, lumbar puncture, parents, pediatric

## Abstract

Background

Lumbar puncture is essential for the diagnosis and treatment of neuroinfections, neuroinflammatory diseases, and central nervous system malignancies. However, some parents refuse to have their children undergo the procedure, causing challenges for accurate diagnoses and choices for the best course of action. This study aimed to evaluate the level of knowledge among parents and caregivers in the Western Region of Saudi Arabia about lumbar puncture in the pediatric age group.

Methodology

This cross-sectional study used a validated self-administered questionnaire in the Western Region of Saudi Arabia. Participants included in this study were caregivers and parents over 18 years of age. The data were represented as numbers and percentages. The data were then analyzed and compared using the chi-square test to examine associations between the two variables. P-values of less than 0.05 were considered statistically significant. Statistical analysis was performed using SPSS version 20.0 (IBM Corp., Armonk, NY, USA).

Results

The study included 993 participants from different age categories (18-50 years). The majority of the participants were female (n = 817, 82.3%) and Saudi Arabian (n = 836, 84.2%). Overall, 66.4% of participants were parents, and 33.6% (n = 334) did not have children. Further, 62.7% (n = 632) of respondents had heard about lumbar puncture before, and 35% (n = 122) had children or relatives who had needed this procedure before. The procedure was approved by 35% of the participants and rejected by 65%. Most (n = 187, 82.4%) parents rejected the procedure because of the fear of complications (n = 144, 63.4%). The most common fears associated with the procedure were the risk of paralysis (n = 597, 60.1%), pain (n = 421, 42.4%), chance of infection (n = 296, 29.8%), or even death (n = 270, 27.2%). Most parents who had refused the procedure would change their mind after discussing with the doctor (n = 166, 73.1%), through clarifying with the attending physician about the potential risks of refusing the procedure (n = 84, 50.6%). The knowledge level of the study participants concerning the lumbar puncture procedure was low in the majority of participants (n = 750, 75.5%). Gender, age, having children, or having contact with the health field were significant factors (p < 0.05) affecting the knowledge about the lumbar puncture procedure.

Conclusions

The present study demonstrated insufficient knowledge regarding lumbar puncture in pediatrics. The most common fears of the procedure were the risk of paralysis, followed by pain, the chance of infection, or even death. Gender, age, having children, or having contact with the health field were significant factors affecting the knowledge about the lumbar puncture procedure. A lack of knowledge on lumbar puncture reflects negative beliefs toward the procedure.

## Introduction

Lumbar puncture (LP) (spinal tap) is a procedure performed on the lower back, in which a needle is inserted into the space between two lumbar vertebras to obtain a sample of cerebrospinal fluid (CSF), which surrounds the brain and the spinal cord [1]. CSF is a metabolically active, dynamic body fluid that serves various crucial purposes [2]. It gives the brain buoyancy, lowers functional brain weight by about 75%, and aids in protecting neural tissues from injury. As the central nervous system (CNS) lacks lymphatics, CSF is a vital medium for chemicals and nutrients to nourish intercellular spaces and a storage area for neural metabolites to be released back into the venous circulation. Despite conditions that increase intracranial pressure, an acute contraction in CSF volume can protect intracranial perfusion and maintain adequate cerebral perfusion pressure. CSF has antibacterial qualities that stop bacteria from multiplying and growing [3].

Despite the crucial role CSF analysis plays in diagnosing neuroinfectious, neuroinflammatory, and CNS malignancies, LPs are frequently avoided in sub-Saharan Africa and other settings with limited resources. When performed by qualified personnel, LP is devoid of serious complications. Severe complications such as cerebral herniation or paralysis, pain at the needle insertion site, and a postspinal headache are uncommon or never seen [4,5]. Patients with bacterial meningitis or herpes encephalitis may experience increased morbidity and mortality if antibiotics are not administered promptly [6].

According to a study from Zambia, the causes of the low uptake of LP in Zambia and other regions still need to be clearly understood [7]. Previous studies investigating LP uptake were frequently restricted to surveys of healthy adults or relatives of children with febrile seizures. In these studies, people who refused or said they would probably refuse an LP frequently expressed fears of pain, paralysis, and death [8,9]. Even though anxiety about pain or unfavorable results may not always be unfounded, a lack of knowledge about the procedure and its potential advantages may make people feel more risk-averse about LP [10]. Additionally, healthcare workers (HCWs) play a crucial role in educating patients and their families, particularly regarding the consent process. However, there is little information available on the knowledge and habits of HCWs concerning the utility and completion of LPs. To remove obstacles from the execution of this crucial neurodiagnostic procedure, it is imperative to comprehend both patient and HCW perspectives regarding LP uptake [10].

When infection is a concern, the risks of not having an LP are more significant than those of the procedure itself [11]. LP is essential for both diagnosis and treatment. However, some parents refuse to have their children undergo the procedure, making it challenging to make an accurate diagnosis and choose the best course of action. According to earlier studies, 25% to 44% of parents object when LP is recommended for their children [12,13].

Parental attitudes toward LP may be influenced by beliefs, fears, and misconceptions [14]. To overcome parental opposition to the procedure, it may be helpful to understand the parental attitudes and knowledge toward LP. A recent Saudi study showed a direct association between a parent’s knowledge of LPs, perception of LPs, and level of education. The less they knew regarding LPs, the more negative they perceived the procedure, with gender and level of education affecting the outcome [15]. Hence, this study aimed to evaluate the level of knowledge among parents and caregivers in the Western Region of Saudi Arabia about LP in the pediatric age group.

## Materials and methods

This cross-sectional study aimed to assess caregivers’ and parents’ level of knowledge, attitude, and perception toward LP. The study used a validated self-administered questionnaire in the Western Region of Saudi Arabia. Participants addressed in this study were caregivers and parents over 18 years of age residing in the Western Region of Saudi Arabia. Eligible patients were children presenting with symptoms suggestive of CNS infection, such as but not limited to meningitis and encephalitis. Participant demographics, including education and area of residence, were collected.

The questionnaire was adopted from another study conducted in the Eastern Region of Saudi Arabia by Alnajim et al. [16]. The questionnaire was modified for this study. Thus, it was revalidated with a Cronbach’s alpha value of 0.718. The data were collected by a self-administered questionnaire comprising four sections and distributed through convenience sampling. The first section consisted of the parents’ demographic data. The second and third sections assessed the level of knowledge and perception about LP. The questions were answered as “yes,” “no,” or “I do not know.” The final section identified the parents’ attitude toward LP. The questions in this section were answered on a scale from “strongly agree” to “strongly disagree.” Participants were then given a list of statements about the purpose, risks, and benefits of LP and asked to indicate whether they agreed or disagreed. LP-related practices were also determined by assessing the likelihood of consent to an LP for themselves or a relative, if requested by a doctor. Data were collected regarding whether an LP was recommended during the current admission and whether it was performed. Dates of initial LP recommendation and completion were abstracted from the patient’s chart.

Statistical analysis

The data were represented as numbers and percentages. The data were then analyzed and compared using the chi-square test to examine associations between the two variables. A p-value of less than 0.05 was considered to be statistically significant. Statistical analysis was performed using SPSS version 20.0 (IBM Corp., Armonk, NY, USA).

Ethical considerations

Approval to conduct this study was obtained from the institutional review board (approval number: 544-22). Patient confidentiality was maintained, and no personal information was reported.

## Results

Table 1 shows the demographic characteristics of the included participants. The study included 993 participants from different age categories (18-50 years). The majority of the participants were female (82.3%) and Saudi Arabian (84.2%). The majority of participants had a university education (66.1%), and 19.6% had a secondary education level. Overall, 16% of parents were working in the health sector, and 61.6% had some relatives/friends working in the health sector. Further, 66.4% of participants were parents, and 33.6% did not have children. The most common sources of medical information among the study participants were internet browsing (53.6%), doctors (49.9%), and relatives or friends working in the medical field (49.7%).

**Table 1:** Demographic characteristics of the study participants.

	Description (n = 993)
Gender	
Male	176 (17.7)
Female	817 (82.3)
Age (years)	
18–29	293 (29.5)
30–39	251 (25.3)
40–49	252 (25.4)
50 or more	197 (19.8)
Nationality	
Saudi Arabian	836 (84.2)
Non-Saudi Arabian	157 (15.8)
Educational level	
Below secondary	31 (3.1)
Secondary	195 (19.6)
University	656 (66.1)
Postgraduate	111 (11.2)
Contact with the health field?	
Work in the healthcare sector	159 (16)
A relative/friend works in the health sector	612 (61.6)
No contact with the healthcare sector	222 (22.4)
Do you have children?	
Yes	659 (66.4)
No	334 (33.6)
What is your trusted source for medical information? (multiple answers were given by the participants)	
News	165 (16.6)
friends and relatives	219 (22.1)
Social media	189 (19)
Your doctor	496 (49.9)
A relative or friend working in the medical field	494 (49.7)
Books and newspapers	169 (17)
Internet browsing	532 (53.6)

Most participants (n = 749, 75.4%) had obtained medical information that turned out to be wrong. They verified this information by asking the doctor (70%) or by searching medical websites (48.7%). Overall, 37.3% of participants had not heard of the LP procedure, and 64.9% did not know anyone who had needed this medical procedure (Table 2).

**Table 2:** Knowledge level of the participants about the lumbar puncture procedure.

	Description (n = 993)
Have you ever obtained medical information that turned out to be wrong?	
Yes	749 (75.4)
No	244 (24.6)
If yes, how did you verify it? (n = 749)	
Searching on medical websites	365 (48.7)
Social media	78 (10.4)
Books and newspapers	38 (5.1)
Questioning family and friends	92 (12.3)
Asking the doctor	524 (70)
Have you ever heard of lumbar puncture?	
Yes	623 (62.7)
No	370 (37.3)
Has any of your children or relatives ever needed a lumbar puncture?	
Yes	349 (35.1)
No	644 (64.9)
If the answer is yes, was the procedure approved? (n = 349)	
Yes	122 (35)
No	227 (65)
If the procedure was rejected, what was the reason for the rejection? (n = 227)	
Fear of complications	144 (63.4)
Mistrust of the doctor	22 (9.7)
Unneeded procedure	61 (26.9)
Lumbar puncture was rejected by (n = 227)	
Mother	28 (12.3)
Father	12 (5.3)
Both parents	187 (82.4)

On the other hand, 62.7% of respondents had heard of the LP procedure, and 35% had children or relatives who had needed this procedure before. The procedure was approved for 35% of participants and was rejected for 65%. Most parents (82.4%) rejected the procedure because of the fear of complications (63.4%).

The most common risks of the lumbar puncture procedure, as mentioned by the study participants, were paralysis (60.1%), pain (42.4%), a chance of infection (29.8%), or even death (27.2%). Most of the parents who had refused the procedure were likely to change their minds after discussing with the doctor (73.1%), by clarifying with the attending physician about the potential risks of refusing the procedure (50.6%) (Table 3).

**Table 3:** Beliefs of participants about the lumbar puncture procedure.

	Description (n = 993)
In your personal opinion, what are the risks involved in lumbar puncture?	
Pain	421 (42.4)
Paralysis	597 (60.1)
Infertility	24 (2.4)
Cerebral hernia	210 (21.1)
Death	270 (27.2)
Chance of infection	296 (29.8)
A drop in cerebral pressure	162 (16.3)
Bleeding	237 (23.9)
If the procedure was refused, did you change your mind after discussing it with the doctor? (n = 227)	
Yes	166 (73.1)
No	61 (26.9)
If yes, what was the reason for changing your mind? (n = 166)	
The doctor’s answer to all questions and inquiries related to the procedure	37 (22.3)
Istikharah (a prayer recited by Muslims who seek guidance from God when facing a decision in their life)	81 (48.8)
Fear of increasing the period of hospitalization	21 (12.7)
Clarification by the attending physician about the potential risks of refusing the procedure	84 (50.6)
Nomination of a doctor with more experience to perform the procedure	34 (20.5)
A relative/friend from the medical field asked about the importance of the procedure	17 (10.2)

Table 4 shows that the knowledge level of the study participants about the LP procedure was low in the majority (75.5%).

**Table 4:** Knowledge level of the participants about the lumbar puncture procedure.

	Description (n = 993)
Knowledge level	
High >60%	243 (24.5)
Low <60%	750 (75.5)

Table 5 shows the factors affecting the knowledge level of the study participants about the LP procedure. Gender, age, having children, or having contact with the health field were the significant factors (p < 0.05) affecting the knowledge about the LP procedure.

**Table 5:** Factors affecting the knowledge level of the study participants about the lumbar puncture procedure.

	Knowledge level		P-value
	High	Low	
Gender			
Male	58 (23.9)	118 (15.7)	0.004
Female	185 (76.1)	632 (84.3)	
Age (years)			
18–29	108 (44.4)	185 (24.7)	0.000
30–39	56 (23)	195 (26)	
40–49	46 (18.9)	206 (27.5)	
50 or more	33 (13.6)	164 (21.9)	
Nationality			
Saudia Arabia	206 (84.8)	630 (84)	0.774
Non-Saudia Arabia	37 (15.2)	120 (16)	
Educational level			
Below secondary	6 (2.5)	25 (3.3)	0.526
Secondary	51 (21)	144 (19.2)	
Graduate	154 (63.4)	502 (66.9)	
Postgraduate	32 (13.2)	79 (10.5)	
Do you have contact with the health field?			
Yes, I work in the health sector	70 (28.8)	89 (11.9)	0.000
Yes, I have some relatives/friends who work in the health sector	129 (53.1)	483 (64.4)	
No	44 (18.1)	178 (23.7)	
Do you have children?			
Yes	136 (56)	523 (69.7)	0.000
No	107 (44)	227 (30.3)	

## Discussion

The LP procedure plays an essential diagnostic role, as well as in anesthetic and therapeutic indications in several neurological pathologies. It is a common practice in emergency departments to detect CNS infections, subarachnoid hemorrhage, and inflammatory processes [17,18]. Although it is frequently performed in the pediatric population, there is a 30% refusal rate for LPs from patients’ caregivers. Previous studies exposed a wide variety of reasons for refusing the procedure. Most rejections arose from misconceptions in the population [19,20]. The present study sought to evaluate the level of knowledge among parents and caregivers in the Western Region of Saudi Arabia about LP in the pediatric age group.

The study included 993 participants in different age categories (18-50 years). The majority of the participants were female (82.3%) and Saudi Arabian (84.2%). Overall, 66.4% of participants were parents, and 33.6% had no children. Further, 62.7% of respondents had heard about the LP procedure before, and 35% of them had children or relatives who had needed it before. The procedure was approved for 35% and was rejected for 65% of the participants (Table 1). In terms of beliefs, most parents (82.4%) rejected the procedure because of the fear of complications (63.4%) (Table 2). The most common fears of the procedure were a risk of paralysis (60.1%), pain (42.4%), a chance of infection (29.8%), or even death (27.2%). Most parents who refused the procedure were likely to change their minds after discussing with the doctor (73.1%), by clarifying with the attending physician the potential risks of refusing the procedure (50.6%) (Table 3). Similarly, a Saudi study by Muammar et al. (2022) found that 56.1% of participants had poor perception, 33.7% had an acceptable perception, and 10.2% had an excellent perception. A greater percentage of participants incorrectly perceived that LP causes severe back pain (79.5%), paralysis (62.4%), infertility (58.5%), and urinary incontinence (70.8%). Moreover, parents believed that LP might result in severe complications (83.2%), commonly results in meningitis (70.4%) as a side effect, and can aggravate poliomyelitis (87.9%). They also inaccurately believed that a post-LP headache could be reduced by using a distinctive type of needle (75.9%). The study showed that there was no relationship between the level of education and attitude [21,22]. On the other hand, some researchers assessed parents’ attitudes toward LP and found that parents’ education was directly related to their attitudes [4,11]. Similarly, regarding gender, it was found that attitude was insignificantly related to parents’ gender. On the contrary, previous studies showed that attitude was influenced by gender, as fathers had a more negative attitude than mothers [8,23].

Regarding parents’ knowledge, the majority of the participants (75.5%) lacked knowledge about LP (Table 4). Gender, age, having children, or having contact with the health field were significant factors affecting the knowledge about the LP procedure (Table 5). Previous studies on this issue in different settings have also found that the public lacks appropriate knowledge about LP [20]. A study by Acoglu et al. (2019) found that most parents had no prior knowledge of the LP procedure, and consent to the procedure was based on trust in the doctor and the information on the consent form. The remaining parents had obtained their knowledge, including unfavorable views, as hearsay information from relatives or friends [4]. This study highlighted that parents with higher education exhibited better knowledge and attitudes about LPs. This was previously noted in studies on diabetes mellitus and stroke [23,24]. This supports that educated people are more receptive to health-related topics. Studies have suggested that people with higher education have different decision-making methods and a distinct thinking process [25]. Furthermore, investing in health education is an investment that would notably improve overall community health [26].

This study has some limitations. As this study was conducted in the Western Region of Saudi Arabia, it may not be applicable to all of Saudi Arabia. As this was a cross-sectional study, it is possible that caregivers agreed to LP later in their admission, and we may have underestimated LP uptake in our patient population.

## Conclusions

The present study demonstrated insufficient knowledge regarding LP in the pediatric population. The most common fears of the procedure were a risk of paralysis, followed by pain, a chance of infection, or even death. Gender, age, having children, or having contact with the health field were the significant factors affecting the knowledge about the LP procedure. A lack of knowledge on LP reflects negative beliefs toward the procedure. It is recommended to obtain the confirmation of parents’ and caregivers’ understanding of the procedure. Furthermore, it is essential to raise awareness and knowledge through the internet and in-hospital campaigns to correct the societal image of LPs.
